# Maximization of Learning Speed in the Motor Cortex Due to Neuronal Redundancy

**DOI:** 10.1371/journal.pcbi.1002348

**Published:** 2012-01-12

**Authors:** Ken Takiyama, Masato Okada

**Affiliations:** 1 Graduate School of Frontier Sciences, The University of Tokyo, Complex Science and Engineering, Chiba, Japan; 2 RIKEN Brain Science Institute, Wako, Japan; University College London, United Kingdom

## Abstract

Many redundancies play functional roles in motor control and motor learning. For example, kinematic and muscle redundancies contribute to stabilizing posture and impedance control, respectively. Another redundancy is the number of neurons themselves; there are overwhelmingly more neurons than muscles, and many combinations of neural activation can generate identical muscle activity. The functional roles of this neuronal redundancy remains unknown. Analysis of a redundant neural network model makes it possible to investigate these functional roles while varying the number of model neurons and holding constant the number of output units. Our analysis reveals that learning speed reaches its maximum value if and only if the model includes sufficient neuronal redundancy. This analytical result does not depend on whether the distribution of the preferred direction is uniform or a skewed bimodal, both of which have been reported in neurophysiological studies. Neuronal redundancy maximizes learning speed, even if the neural network model includes recurrent connections, a nonlinear activation function, or nonlinear muscle units. Furthermore, our results do not rely on the shape of the generalization function. The results of this study suggest that one of the functional roles of neuronal redundancy is to maximize learning speed.

## Author Summary

There are overwhelmingly more neurons than muscles in the motor system. The functional roles of this neuronal redundancy remains unknown. Our analysis, which uses a redundant neural network model, reveals that learning speed reaches its maximum value if and only if the model includes sufficient neuronal redundancy. This result does not depend on whether the distribution of the preferred direction is uniform or a skewed bimodal, both of which have been reported in neurophysiological studies. We have confirmed that our results are consistent, regardless of whether the model includes recurrent connections, a nonlinear activation function, or nonlinear muscle units. Additionally, our results are the same when using either a broad or a narrow generalization function. These results suggest that one of the functional roles of neuronal redundancy is to maximize learning speed.

## Introduction

In the human brain, numerous neurons encode information about external stimuli, e.g., visual or auditory stimuli, and internal stimuli, e.g., attention or motor planning. Each neuron exhibits different responses to stimuli, but neural encoding, especially in the visual and auditory cortices, can be explained by the maximization of stimulus information [1]–[3]. This maximization framework can also explain learning that occurs when the same stimuli are repeatedly presented; previous neurophysiological experiments have suggested that perceptual learning causes changes in neural encoding to enhance the Fisher information of a visual stimulus [4]. However, a recent study has suggested that information maximization alone is insufficient to explain neural encoding. Salinas has suggested that “how encoded information is used” needs to be taken into account: neural encoding is influenced by the downstream circuits and output units to which neurons project, and it is ultimately influenced by animal behavior [5]. In the motor cortex, neural encoding is influenced by the characteristics of muscles (output units) because motor cortex neurons send motor commands to muscles through the spinal cord. In adaptation experiments, some motor cortex neurons exhibit rotations in their preferred directions (PDs), and these rotations result in a population vector that is directed toward a planned target [6]. Neural encoding therefore changes to minimize errors between planning and behavior, suggesting that neural encoding is influenced by behavior and properties of output units.

A critical problem exists in the relationship between motor cortex neurons and output units: the neuronal redundancy problem, or overcompleteness, which refers to the fact that the number of motor cortex neurons far exceeds the number of output units. Many different combinations of neural activities can therefore generate identical outputs. Neurophysiological and computational studies have revealed that the motor cortex exhibits neuronal redundancy [7], [8]. However, it remains unknown how neuronal redundancy influences neural encoding. In other words, we do not yet understand the functional roles of neuronal redundancy in motor control and learning, though other types of redundancies are known to play various functional roles [9].

One of these types of redundancy is muscle redundancy: many combinations of muscle activities can generate identical movements. The functional roles of this muscle redundancy include impedance control to achieve accurate movements [10], reduction of motor variance by constructing muscle synergies [11], and learning internal models by changing muscle activities [12]. Another redundancy is kinematic redundancy: many combinations of joint angles result in identical hand positions. This redundancy ensures the stability of posture even if one joint is perturbed [13], and it facilitates of motor learning by increasing motor variance in a dimension irrelevant to the desired movements [14]. Redundancies therefore play important functional roles in motor control and learning.

Similar to the muscle and kinematic redundancies, neuronal redundancy likely has functional roles in motor control and learning. However, the functional roles of this redundancy are unclear. Here, using a redundant neural network, we investigate these functional roles by varying the number of model neurons while holding the number of output units constant. This manipulation allows us to control the degree of neuronal redundancy because, if a neural network includes a large number of neurons and a small number of output units, many different combinations of neural activities can generate identical outputs. It should be noted that we used a redundant neural network model that can explain neurophysiological motor cortex data [7]. The key conclusion arising from our study is that one of the functional roles of neuronal redundancy is the maximization of learning speed.

Initially, a linear model with a fixed decoder was used. Analytical calculations revealed that neuronal redundancy is a necessary and sufficient condition to maximize learning speed. This maximization is invariant whether the distribution of PDs is unimodal [6] or bimodal [15]–[17]; both distributions have been reported in neurophysiological investigations. Second, numerical simulations confirmed the invariance of our results, even when the neural network included an adaptable decoder, a nonlinear activation function, recurrent connections, or nonlinear muscle units. Third, we show that our results do not depend on learning rules by using weight and node perturbation, both of which are representative stochastic gradient methods [18]. Finally, we demonstrate that our hypothesis does not depend on the shape of the generalization function which shape depends on the task (broad or sharp in force field [19], [20] or visuomotor rotation adaptation [21], respectively). Our results strongly support our hypothesis that neuronal redundancy maximizes learning speed.

## Results

**Figure 1:**
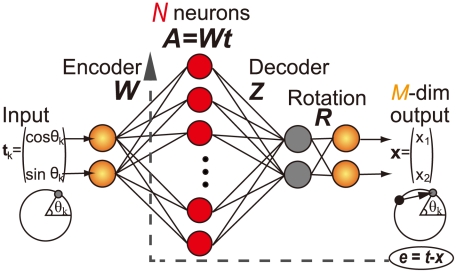
Graphical model of a redundant neural network.

Neuronal redundancy is defined as the dimensional gap between the number of neurons 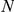 and the number of outputs 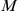. It is synonymous with overcompleteness [22]: many combinations of neural activities 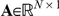 can generate identical outputs 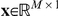 through a decoder 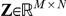 (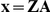) because there are more neurons than necessary, i.e., 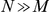 (Figure 1). It should be noted that neuronal redundancy is defined not by 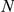 but by the relationship between 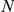 and 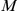. In most parts of this study, the number of constrained tasks 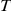 is the same as 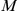 and is set to two, i.e., 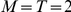, so there is neuronal redundancy if 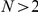. Thus, throughout this paper, the extent of neuronal redundancy can be expressed simply using the number of neurons. In this study, we can change only the neuronal redundancy; 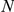 can be increased while 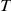 is held constant at two, enabling the investigation of the functional roles of neuronal redundancy. In the *Importance of Neuronal Redundancy* section, we distinguish the effects of neuronal redundancy from the effects of neuron number by varying both 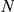 and 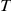.

In this study, we discuss the relationship between neuronal redundancy and learning speed by assuming adaptation to either a visuomotor rotation or a force field. These tasks are simulated by using a rotational perturbation 
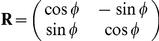
 where 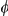 is the rotational angle. Due to this perturbation, if an error occurs between target position 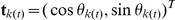 and output (motor command) 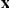 in the 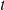
th trial, neural activities 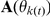 are modified to minimize the error, where 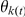 is the angle of the 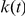
th target which is radially and equally distributed (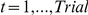, 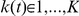, 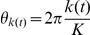). To model the learning process in the motor cortex, we used a linear rate model, which can reproduce neurophysiological data [7] and be easily analyzed. In this model, 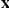 is given by a weighted average of 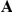, and each component of 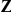 is accordingly set to 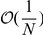, i.e., 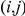
th component of 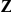 is defined as 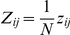, where 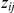 is a variable that is independent of 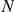. Because of this assumption, the learning rate is set to 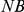 such that the trial-to-trial variation of 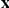 do not depend on 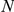 (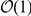), but the optimized learning rate 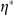 is 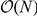 (see Text S1), i.e., 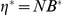, suggesting that we consider the quasi-optimal learning rate in this study. It should be noted that, because the following results do not depend on 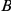, our results hold when the optimal learning rate is used. Furthermore, even when each component of 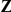 is 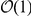, the following results are invariant if we set the learning rate to its optimal value (see Text S1). Our study shows that neuronal redundancy is necessary and sufficient to maximize learning speed.

### Neuronal redundancy maximizes learning speed

#### Fixed homogeneous decoder

In the case of a fixed decoder, 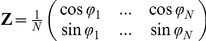, the 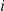
th neuron has uniform force amplitude (FA) (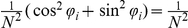) and force direction (FD), 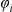, which is randomly sampled from a uniform distribution. Because of its uniformity, we refer to this decoder as a fixed homogeneous decoder. This model corresponds to the one proposed by Rokni et al. [7].

In this case, the squared error can be calculated recursively as
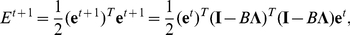
(1)where 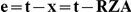. Here, we assume that a single target is repeatedly presented for simplicity (general case is discussed in the *Methods* section), 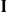 is the identity matrix, 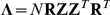, 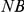 is the learning rate, and neural activity 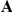 is updated as
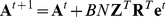
(2)for the 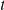
th trial to minimize the squared error. Multiplication by 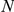 in equation (2) is included for the purpose of scaling; it ensures that the amount of trial-to-trial variation in 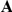 does not explicitly depend on 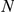. Equation (1) can thus be simplified as
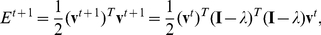
(3)where the diagonal elements of 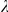, 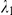 and 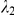, are eigenvalues of 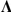, 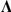 is decomposed as 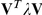 (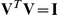), 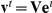, and learning speed is therefore determined based on the eigenvalues of
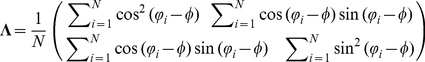
(4)each component of which is 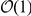. The larger 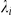 becomes, the faster learning becomes (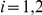). It should be noted that learning speed and 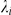 do not explicitly depend on 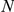.

Analytical calculations can yield necessary and sufficient conditions to maximize learning speed (see the *Methods* section). The following self-averaging properties [23] maximize learning speed or maximize the minimum eigenvalue of 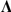:
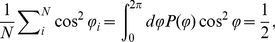
(5)

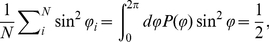
(6)and
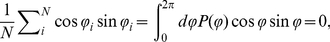
(7)where 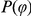 is the probability distribution in which FDs are randomly sampled. It remains unknown what kind of conditions can satisfy the self-averaging properties. The self-averaging properties are satisfied if and only if the neural network model includes sufficient neuronal redundancy. In other words, learning speed is maximized if and only if 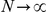. If the neural network includes neuronal redundancy, the self-averaging properties exist. Conversely, if the self-averaging properties exist, the neural network model should include sufficient neuronal redundancy because Monte Carlo integration shows a fluctuation of 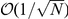 [24]. Thus, in the case of a fixed homogeneous decoder, neuronal redundancy plays a functional role in maximizing learning speed.

**Figure 2:**
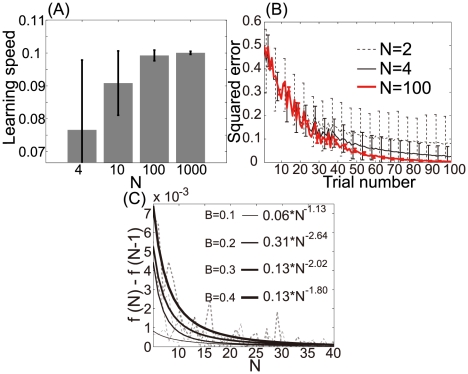
Relationship between learning speed and neuronal redundancy (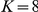). (A): Learning speed when 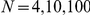, or 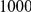. The bar graph and error bars depict sample means and standard deviations, both of which are calculated using the results of randomly sampled sets of 1000 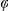 values. (B): Learning curves when 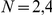, or 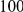. These curves and error bars show averaged values and standard deviations of errors. (C): Relationship between learning speed and the number of model neurons when 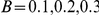, or 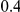. The horizontal axis represents the number of neurons 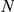 and the vertical axis represents 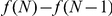, where 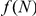 is the learning speed when the number of neurons is 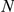. Dotted and solid lines denote the average learning speed and power functions fitted to the values, respectively.

We numerically confirmed the above analytical results. Figures 2A and 2B show the learning speed and learning curves calculated using the results of 1,000 sets of randomly sampled 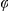 values, an identical target sequence (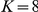), and 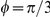. The more neuronal redundancy grows, the faster learning speed becomes. Figure 2C shows the relationship between learning speed and neuronal redundancy. The horizontal axis denotes the number of neurons, and the vertical axis denotes the increase in learning speed. Although a saturation of the increase can be seen, greater neuronal redundancy still yields faster learning speed. Therefore, these figures support our analytical results: in the case of a fixed homogeneous decoder, neuronal redundancy maximizes learning speed.

#### Fixed non-homogeneous decoder

**Figure 3:**
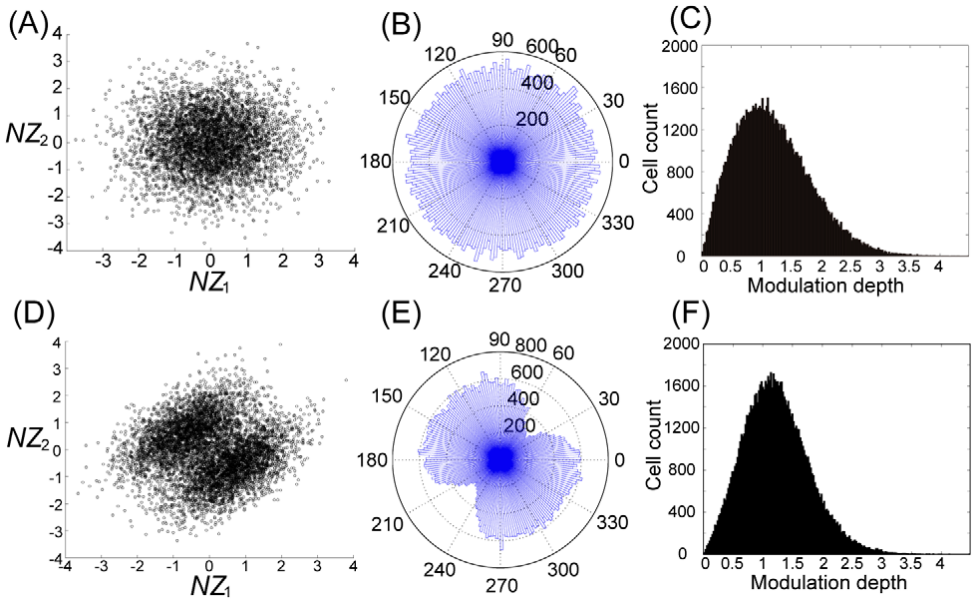
Network properties when 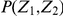 satisfies equations (8) and (9). (A): Scatter plot of 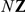 when 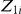 and 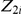 are randomly sampled from a unimodal Gaussian distribution (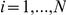). (B), (C): Histogram of preferred direction and modulation depth when 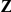 is randomly sampled as shown in (A). (D): Scatter plot of 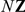 when 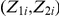 are randomly sampled from a bimodal Gaussian distribution. (E), (F): Histograms of preferred direction and modulation depth when 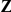 is randomly sampled as shown in (D).

The question remains whether it is necessary for FD and FA to be distributed uniformly, so we assume that the values 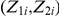 are randomly sampled from the probability distribution 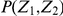 to make FD and FA non-homogeneous, i.e., FDs are non-uniformly distributed, and FAs are different for each neuron. In the case of a non-homogeneous decoder, the necessary and sufficient conditions to maximize learning speed are also the following self-averaging properties:
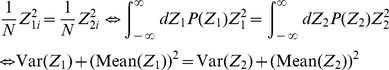
(8)and
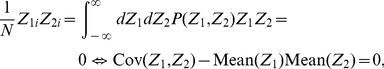
(9)where 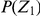 and 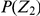 are marginalized distributions. Figures 3A and 3D show distributions of 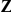 that satisfy equations (8) and (9). 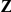 is randomly sampled from unimodal and bimodal Gaussian distributions in Figures 3A and 3D, respectively. Because these figures show the non-uniformity in both FD and FA, neuronal redundancy maximizes learning speed regardless of these non-uniformities.

#### Distribution of preferred directions

Some neurophysiological studies have suggested that the distribution of PD is a skewed bimodal [15]–[17], but other neurophysiological studies have suggested that the distribution of PD is uniform [6]. We investigated whether our results were consistent with the results of these neurophysiological studies. Figures 3B and 3E depict the distribution of preferred directions (PDs) that results when 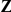 is randomly sampled as shown in Figures 3A and 3D, respectively, with PDs calculated as 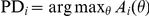 (see the *Methods* section). Figures 3B and 3E show that both a skewed bimodal distribution and a uniform distribution can be observed when 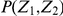 satisfies equations (8) and (9), suggesting that our hypothesis is consistent with the results of previous neurophysiological experiments.

Figures 3C and 3F show the distribution of modulation depth, which is calculated as 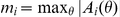 (see the *Methods* section). Our results suggest that the distribution of modulation depth is skewed.

#### Adaptable decoder

**Figure 4:**
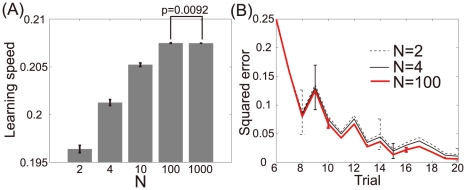
Relationship between learning speed and neuronal redundancy when the decoder is adaptable (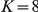). (A): Bar graphs and error bars depict sample means and standard deviations both of which are calculated using the results from 1000 sets of 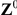 values. (B): Learning curves when 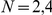, or 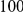. These curves and error bars show averaged values and the standard deviations of the errors.

We have analytically elucidated the relevance of neuronal redundancy to learning speed only when 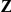 is fixed, but the question remains of whether neuronal redundancy can maximize learning speed even when 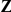 is adaptable. In this case, it is analytically intractable to calculate learning speed, so we used numerical simulations. Figure 4A shows the learning speed when 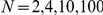, or 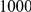 in the case of an adaptable decoder. Although there was no significant difference in learning speed between the cases in which 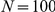 and 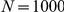, neuronal redundancy maximized learning speed even if the decoder was adaptable. Figure 4B, which shows the learning curve when 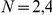, or 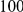, also supports the maximization.

### Importance of neuronal redundancy

Although we have revealed that neuronal redundancy maximizes learning speed when 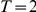, it is important to verify that the effect is caused by the neuronal redundancy, i.e., the dimensional gap between 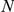 and 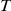, and not simply the number of neurons 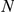. In this section, we investigate this question by varying both 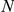 and 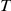 while assuming that each component of 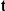 is randomly sampled from a Gaussian distribution.

**Figure 5:**
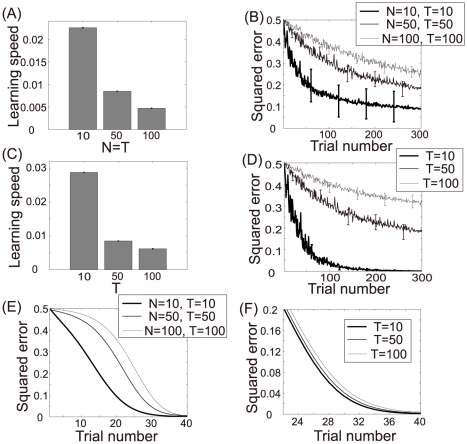
Importance of neuronal redundancy (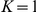). (A): Learning speed when 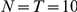, 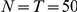, or 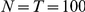, where N and T are the number of neurons and constrained tasks, respectively. The bar graphs and error bars depict the sample means and standard deviations, both of which are calculated using the results of 1000 sets of 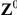 values. (B): Learning curves when 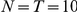, 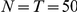, or 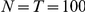. These curves and error bars show the average values and the standard deviations of the errors. (C): Learning speed when 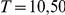, or 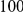, and 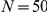. The bar graphs and error bars depict the sample means and the standard deviations, both of which are calculated using the results of 1000 sets of 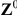 values. (D): Learning curves when 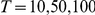, and 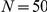. These curves and error bars show the average values and the standard deviations of the errors. (E): Learning curves calculated when 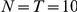, 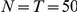, or 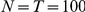 and decoder 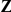 is adaptable. (F): Learning curves calculated when 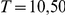, or 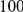; 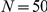; and the decoder 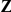 is adaptable.

Figures 5A and 5B show the learning speed and the learning curve produced when 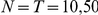, and 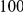 with a fixed non-homogeneous decoder. If 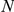 alone were important for maximizing learning speed, learning speed would be faster when 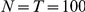 than when 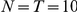 or 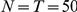. However, the results shown in these figures support the opposite conclusion, i.e., learning speed becomes slower when 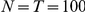 compared to the other cases. This result suggests that the number of neurons alone is not important for maximizing learning speed.

Figures 5C and 5D show the learning speed and learning curve produced when 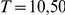, or 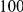 with 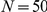 and a fixed non-homogeneous decoder. If neuronal redundancy were important, the learning speed would be faster when 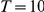 than when 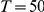 or 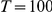. These figures support this hypothesis; learning speed increased when 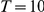 compared to the other cases. Taken together, these results indicate that the important factor for maximizing learning speed is in fact neuronal redundancy and not simply the number of neurons.

In addition, we investigated whether neuronal redundancy or neuron number is important when 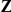 is adaptable. In this case, we only show learning curves because learning speed cannot be exponentially fitted, which makes it impossible to calculate learning speed. Figures 5E and 5F show the learning curves calculated when 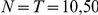, or 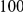 and 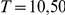, or 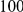 with 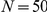. These figures show the same results as the case when 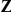 is fixed; even when 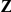 is adaptable, the important factor for maximizing learning speed is neuronal redundancy, not simply the number of neurons.

### Generality of our results

The generality of our results should be investigated because we analyzed only linear and feed-forward networks, but neurophysiological experiments have suggested the existence of recurrent connections [25] and nonlinear neural activation functions [26]. Also, only a linear rotational perturbation task was considered, so we need to investigate whether our results hold when the constrained tasks are nonlinear because, in fact, motor cortex neurons solve nonlinear tasks. The neurons send motor commands and control muscles whose activities are nonlinearly determined: muscles can pull but cannot push. Using numerical simulations, we show that neuronal redundancy maximizes learning speed, even when the neural network includes recurrent connections (Figure S1), when it includes nonlinear activation functions (Figure S2), and when the task is nonlinear (Figure S3).

In addition, we used only deterministic gradient descent, so the generality regarding the learning rule needs to be investigated. In fact, previous studies have suggested that stochastic gradient methods are more biologically relevant than deterministic ones [27], [28]. Analytical and numerical calculations confirm that our results are invariant even when the learning rule is stochastic (Figure S4). Our results therefore have strong generality.

#### Activity noise and plasticity noise

Although our results have strong generality, there is still an open question regarding the robustness of noise: does neuronal redundancy maximize learning speed even in the presence of neural noise? Actually, neural activities show trial-to-trial variation [29], and the neural plasticity mechanism also includes trial-to-trial fluctuations [7]. This section investigates the relationships between neuronal redundancy, learning speed, and neural noise.

**Figure 6:**
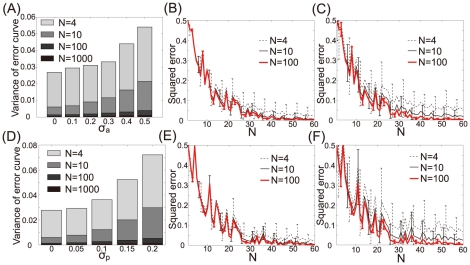
Relationship between neuronal redundancy and neural noise (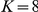). (A): Variance of the learning curve when 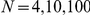, or 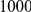 and 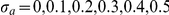. The bar graphs show the average values of randomly sampled sets of 1000 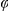 values. (B): Learning curves calculated when 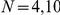, or 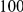, and 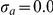. These curves and error bars show the average values and the standard deviations of the errors. (C): Learning curves calculated when 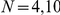, or 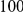, and 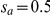. (D): Variance of the learning curve when 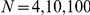, or 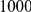 and 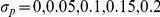. (E): Learning curves when 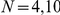, or 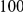, and 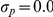. (F): Learning curves calculated when 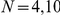, or 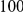, and 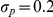.

Figures 6A and 6D show the variance of learning curves when 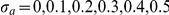 and 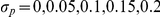, respectively, with 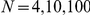, or 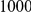 and 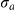 and 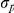 representing the standard deviations of activity noise and plasticity noise, respectively. The definition of the variance is 
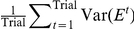
, which is a measure of the stability of learning. Examples of learning curves are shown in Figures 6B, 6C, 6E, and 6F. These figures show that neuronal redundancy enhances the stability of learning by eliminating the influences of activity and plasticity noise. Neuronal redundancy therefore not only maximizes learning speed but also facilitates robustness in response to neural noise.

#### Shape of the generalization function

In many situations, learning in one context is generalized to different contexts, such as different postures [30], different arms [31], and different movement directions [19]–[21], with the degree of generalization depending on the task. In this study, we define the generalization function as the degree of generalization to different movement directions. The performance of reaching towards 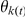 is generalized to that of reaching towards 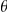, and the degree of this generalization is determined by the generalization function 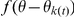. In visuomotor rotation adaptation, the generalization function is narrow in the direction metric [21]. In contrast, the generalization function is broad in force field adaptation [19], [20]. To investigate the generality of our results with respect to various kinds of tasks, it is necessary to investigate the relationships between neuronal redundancy, learning speed, and the shape of the generalization function.

**Figure 7:**
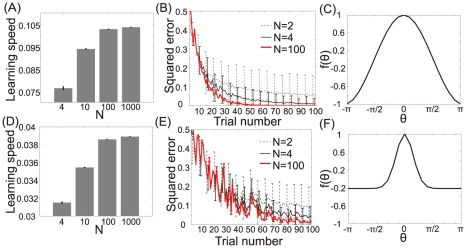
Relationship between neuronal redundancy, learning speed, and the shape of the generalization function (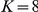). (A): Learning speed when 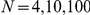, or 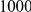, and 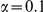. The bar graphs and error bars depict sample means and standard deviations, both of which are calculated using the results of randomly sampled sets of 1000 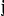 values in the case of a broad generalization function. (B): Learning curves calculated when 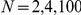, and 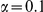. These curves and error bars show the average values and standard deviations of the errors. (C): The generalization function with 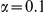. (D): The learning speed when 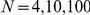, or 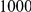, and 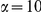. Bar graphs and error bars depict the sample means and standard deviations when the generalization function is narrow (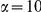). (E): Learning curves calculated when 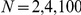, and 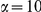. (F): The generalization function with 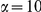.

Figure 7 shows the relationship between the shape of the generalization function and learning speed. Figures 7A and 7B show the learning speed and learning curve calculated when the generalization function is broad (Figure 7C). Figures 7D and 7E show the learning speed and learning curve calculated when the generalization function is narrow (Figure 7F). Although these figures show that narrower generalization results in a slower learning speed, neuronal redundancy maximizes learning speed independently of the shape of the generalization function.

## Discussion

We have quantitatively demonstrated that neuronal redundancy maximizes learning speed. The larger the dimensional gap grows between the number of neurons and the number of constrained tasks, the faster learning speed becomes. This maximization does not depend on whether the PD distribution is unimodal or bimodal, the decoder is fixed or adaptable, the network is linear or nonlinear, the task is linear or nonlinear, or the learning rule is stochastic or non-stochastic. Additionally, we have shown that neuronal redundancy has another important functional role: it provides robustness in response to neural noise. Furthermore, neuronal redundancy maximizes learning speed in a manner independent of the shape of the generalization function. These results strongly support the generality of our results.

Neuronal redundancy maximizes learning speed because only 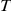 equalities, 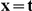, need to be satisfied, and 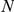-dimensional neural activity 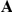 is adaptable (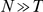). This dimensional gap yields the large 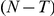 dimensional subspace of 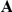 in which the 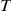 equalities are satisfied. The more 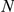 increases, the greater the fraction of the subspace becomes: 
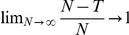
. Neuronal redundancy, rather than the number of neurons, thus enables 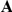 to rapidly reach a single point in the subspace. This interpretation likely applies even in the cases of an adaptable decoder, recurrent connections, a nonlinear network, a nonlinear task, and a stochastic learning rule. Furthermore, this interpretation is supported by the results shown in Figure 5; the bigger 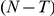 grows, the faster learning speed becomes.

At first glance, our results may seem inconsistent with the results of Werfel et al. [18], who concluded that learning speed is inversely proportional to 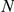. In their model, because they considered the single-layer linear model, 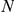 is the same as the number of input units, which is defined as 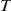
( = 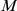) in the present study. A similar tendency can be observed in Figure 5; the more 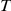 increases, the slower learning speed becomes. We calculated the optimal learning rate and speed as shown in Text S1, and confirmed that learning speed is inversely proportional to 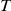. Thus, our results are consistent with Werfel's study and additionally suggest that neuronal redundancy maximizes learning speed.

Neuronal redundancy plays another important role: generating robustness in response to neural noise (Figure 6). Because neuronal redundancy has the same meaning as overcompleteness, its functional role is the same as the robustness of overcompleteness in the face of perturbations in signals [32]. This additional functional role further supports our hypothesis that neuronal redundancy is a special neural basis on which to maximize learning speed. For example, if we increase the learning rate 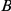 in a non-redundant network, the learning speed approaches the maximal speed in a redundant network in which the learning rate is fixed to 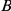. As shown in Figure 6, however, a non-redundant network is not robust with respect to neural noise. Furthermore, neuronal redundancy minimizes residual errors when the neural network includes synaptic decay [7] (see the *Methods* section and Figure S5). Thus, neuronal redundancy represents a special neural basis for maximizing learning speed while minimizing residual error and maintaining robustness in response to neural noise.

## Methods

### Model definition

Our study assumed the following task: participants move their arms towards one of 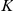 radially distributed targets. If the 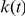
th target is presented in the 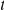
th trial, the neural network model receives the input 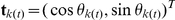 (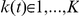, 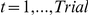), where 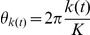. The input units project to neurons (hidden units), the activities of which are determined by
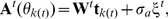
(10)where 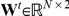 is synaptic weight in the 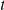
th trial, 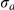 is the standard deviation of neural activity noise, 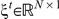 denotes independent normal Gaussian random variables, and 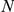 is the number of neurons (Figure 1). The 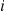
th neuron has a PD given by 
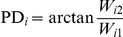
 and a modulation depth 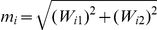, where 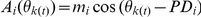, this cosine tuning having been reported by many neurophysiological studies.

The neural population generates a force of 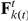 through a decoder matrix 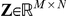:
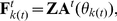
(11)where 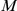 is the number of outputs, which, in most cases, is set to 2. When 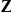 is fixed and homogeneous, the 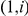
th and 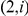
th components of 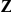 are defined as 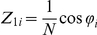 and 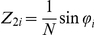, respectively, where division by 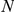 is used for scaling and FD 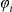 is randomly sampled from a uniform distribution (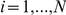). When 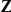 is fixed and non-homogeneous, 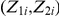 is randomly sampled from a probability distribution 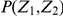 and divided by 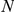. As a result, the neural network generates a final hand coordinate 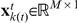:
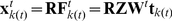
(12)which means that 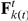 is perturbed by a rotation 
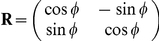
 which assumes a visuomotor rotation or curl force field. Rotational perturbations are assumed because many behavioral studies have used them. Because we discuss only the endpoint of the movement, we refer to 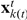 as the motor command. The constrained tasks are those that the neural network generates 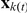 toward 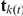, i.e., 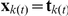, which means the number of constrained tasks 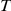 is the same as 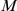. We used 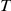 instead of 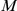 in the following sections.

If the error occurs between 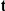 and 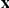, synaptic weights 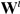 are adapted to reduce the squared error, which is defined as 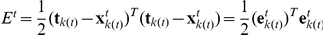, based on a gradient descent method
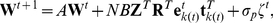
(13)where 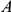 is the synaptic decay rate, 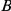 is the learning rate (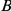 is set to 0.2 in most parts of the present study), 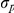 is the strength of synaptic drift, and 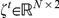 denotes normal Gaussian random variables. Since each component of 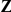 is 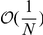, multiplying 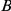 by 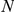 allows trial-by-trial variation of both 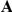 and 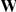 to be 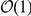. As shown in Text S1, the optimal learning rate 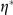 is 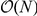 (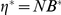), suggesting that we consider a quasi-optimal learning rate. It should be noted that our results hold whether the learning rate is optimal or quasi-optimal because the results do not depend on 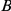. It should also be noted that the amount of variation in 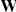 does not explicitly depend on 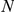.

### Learning curve

Equation (13) yields the following update rule of squared error:
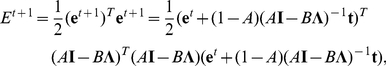
(14)where 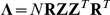, and 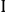 denotes the identity matrix. At first, we assume a case in which 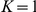 for simplicity. Because 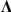 is symmetric, 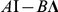 can be decomposed as 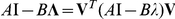, where each row of 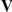 is one of the eigenvectors (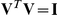) and each diagonal component of a diagonal matrix 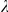 is one of the eigenvalues of 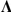. This decomposition transforms equation (14) into the simple form
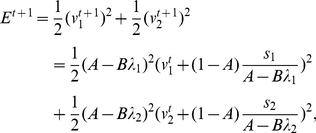
(15)where 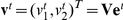 and 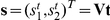. This recurrence formula yields the analytical form of the learning curve:
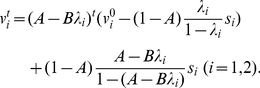
(16)


Equation (16) requires that the larger the eigenvalues become, the faster the learning speed becomes and the smaller the residual error becomes (Figure S5). Because
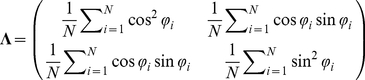
(17)whose component is 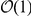, simple algebra gives the analytical form of the eigenvalues,
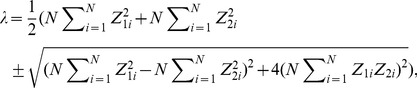
(18)which are also 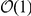, suggesting that learning speed does not depend explicitly on 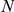. Because the learning speed is determined by the smaller eigenvalue, the necessary and sufficient conditions to maximize learning speed, or to maximize the smaller eigenvalue, are
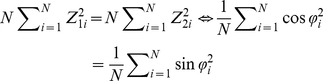
(19)and
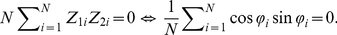
(20)


What kind of conditions can simultaneously satisfy equations (19) and (20)? The only answer is sufficient neuronal redundancy, i.e., 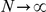, because sufficient neuronal redundancy enables self-averaging properties to exist in a neural network, i.e.,
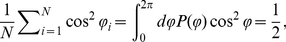
(21)

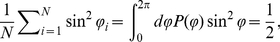
(22)and
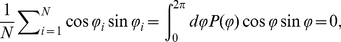
(23)where 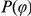 is the probability distribution in which FDs are randomly sampled. Conversely, if equations (21), (22), and (23) are satisfied in all of the sets of randomly sampled FDs, the number of neurons needs to satisfy 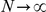 because the fluctuation of Monte Carlo integrals is 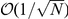 [24]. Therefore, to maximize learning speed, the necessary and sufficient condition is sufficient neuronal redundancy.

The above analytical calculations hold even when 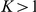. Equation (13) yields the recurrence equation of the squared error:
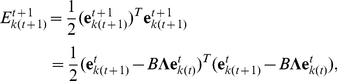
(24)where 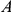 is set to 1 for simplicity. Using 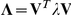, this equation can be written as
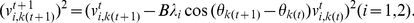
(25)The larger the eigenvalue becomes, the faster learning speed becomes if 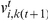 and 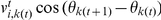 have the same sign, or if 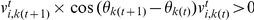. This inequality is appropriate if the equality 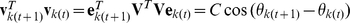 can be proved, where 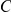 is a positive constant. To prove this equality, let us assume that in the 1st trial after the rotational perturbation 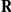 is applied, output can be written as 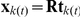 because the neural network can generate accurate outputs if there is no perturbation. In this case,
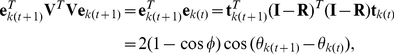
(26)where 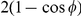 is a positive constant. Thus, the larger 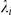 becomes, the faster learning speed becomes even when 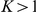; analytical calculations show that neuronal redundancy maximizes learning speed even when 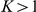.

### Fixed non-homogeneous decoder

When 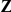 is fixed and non-homogeneous, i.e., 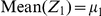, 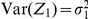, 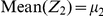, 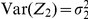, and 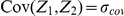, the necessary and sufficient conditions for maximizing learning speed are given by the following equations:
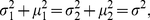
(27)

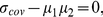
(28)with neuronal redundancy assumed. Equations (27) and (28) can be satisfied when, for example,
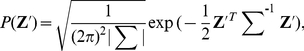
(29)(shown in Figure 3A with 
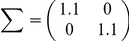
 and 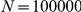), or
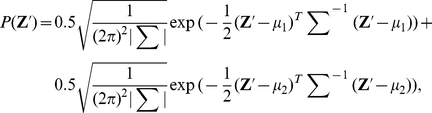
(30)(shown in Figure 3D with 
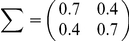
, 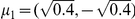, 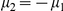 and 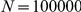), where 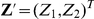. Because the learning rate of motor commands is determined by 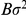 (see the following section), 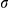 is determined based on the results of behavioral studies [33]. We cannot analytically calculate the general class of 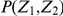 and the distributions of PDs satisfying equations (27) and (28), but broad classes of those distributions can satisfy these equations because the classes include even asymmetric distributions, e.g., when 
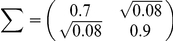
, 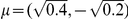.

### Learning rule of decoder 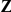


When 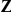 is adaptable, this is also adapted to minimize the squared error:
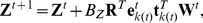
(31)where 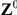 is set to 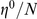, 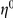 is a normal Gaussian random variable, and 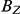 is set to 0.1 in the Adaptable Decoder section and 0.05 in the Importance of Neuronal Redundancy section. This learning rule corresponds to back-propagation [34].

### High dimensional tasks

In the Importance of Neuronal Redundancy section, the neural network generates the output 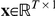, which is determined by
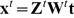
(32)for the 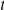
th trial. An initial value of 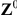 is randomly sampled from the normal Gaussian distribution and divided by 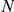 for scaling. The input 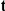 is randomly sampled from the normal Gaussian distribution and is normalized to satisfy 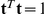 to avoid the effect of this value on learning speed. In addition, we used a fixed value of 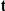 because the generalization function (see the following section) strongly depends on 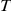, i.e., 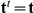. It should be noted that learning speed does not explicitly depend on 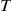 because learning speed is determined only by the minimum eigenvalue of 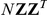.

### The generalization function and the update rule for motor commands

Equation (13) yields the following update rule for motor commands:
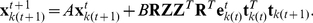
(33)If equations (27) and (28) (or (22) and (23)) are satisfied, equation (33) can be written as
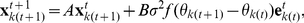
(34)where the cross term of 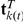 and 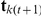 determines the generalization function 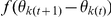, e.g., 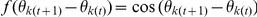, if we define 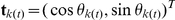. We set 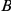 and 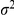 to satisfy 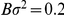. It should be noted that equation (34) corresponds to a model for sensorimotor learning that can explain the results of behavioral experiments [35], suggesting that our hypothesis is consistent with the results of behavioral experiments.

Because the shape of the generalization function depends on the task, we need to confirm the generality of our results with regard to the shape of the generalization function. To simulate various shapes of generalization functions, we used the von-Mises function
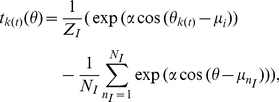
(35)where 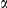, 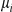, and 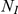 are the precision parameter, the preferred direction of the 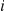
th input unit, and the number of input units, respectively. The normalization factor 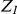 is determined to make 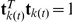 to avoid the influence of this value on the learning speed, where 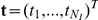. This normalization permits us to investigate the influence of the shape of the generalization function alone on learning speed. The larger the value of 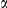, the sharper the shape of the generalization function becomes. We set 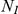 to 100 throughout this study.

### Numerical simulation procedure

We conducted 100 baseline trials with 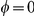 and 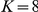 to identify the baseline values of 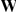. The initial value of 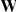, 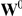, was set to 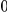. After these trials, 100 learning trials were conducted using 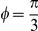 and 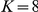. Learning speed 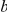 was calculated by fitting the exponential function 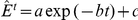 to 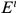. All the figures denote 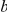 which was obtained only in learning trials. The present study calculated learning speed and learning curves by averaging the results of 1000 sets of baseline and learning trials, each set including an identical target sequence that was randomly sampled, and each set using different FD values.

For all of the statistical tests, we used the Wilcoxon sign rank test. It should be noted that the 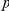-value was indicated only if the value was significantly different from 0; no statistically significant differences were detected.

## Supporting Information

Figure S1
**Relationship between learning speed, neuronal redundancy, and adaptable recurrent connections (** 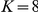 **).** (A): Learning speed when 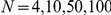 and 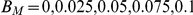. The whiter the color, the faster the learning speed. (B): Learning curves obtained when N = 10, 50, or 100 and 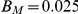. These curves show the average values of 1,000 randomly sampled sets of 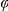. Error bars represent the standard deviations of the errors. (C): Learning curves obtained when 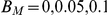 and 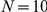. These curves and error bars show average values and standard deviations. (D): Variance of the learning curve when 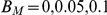 and 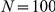 (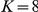). These variances are average values from 1,000 randomly sampled sets of 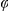.(EPS)

Figure S2
**Relationship between learning speed and neuronal redundancy in the case of a nonlinear neural network (** 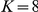 **).** (A): Learning speed when N = 10, 50, 100, and 1000. The bar graphs and error bars depict sample means and standard deviations, both of which are calculated using the results of 1,000 randomly sampled sets of 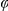 values. (B): Learning curves obtained when 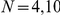, or 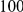. These curves and error bars show average values and the standard deviations of the errors.(EPS)

Figure S3
**Relationship between learning speed and neuronal redundancy when the neural network includes nonlinear muscle units (** 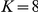 **).** (A): The bar graphs and error bars depict sample means and standard deviations, both of which were calculated using the results of 1,000 randomly sampled sets of 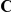 values. (B): Learning curves obtained when 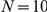 or 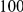. These curves and error bars show average values and the standard deviations of the errors.(EPS)

Figure S4
**Relationship between learning speed and neuronal redundancy in the case of weight perturbation and node perturbation (** 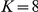 **).** (A): Learning speed when 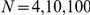, or 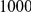, with weight perturbation as the learning rule. The bar graphs and error bars depict sample means and standard deviations, both of which are calculated using the results of 1,000 randomly sampled sets of 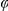. (B): Learning curves obtained when 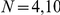, or 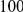, with weight perturbation as the learning rule. These curves and error bars show the average values and the standard deviations of the errors. (C): Learning speed when 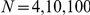, or 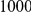, with node perturbation as the learning rule. The bar graphs and error bars depict sample means and standard deviations, both of which are calculated using the results of 1,000 randomly sampled sets of 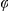. (D): Learning curves obtained when 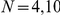, or 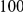, with node perturbation as the learning rule. These curves and error bars show average values and the standard deviations of the errors.(EPS)

Figure S5
**Relationship between residual error, learning speed, and neuronal redundancy with synaptic decay included (** 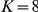 **).** (A): Residual error when 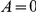. The bar graphs and error bars denote sample means and standard deviations, both of which are calculated using the results of 1,000 randomly sampled sets of 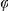 values. (B): Learning speed when 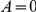. The bar graphs and error bars depict sample means and standard deviations. (C): Learning curves obtained when 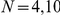, and 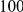 and 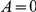. These curves and error bars show average values and standard deviations. (D): Residual error when 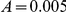. (E): Learning speed when 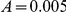. (F): Learning curve when 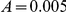. (G): Residual error when 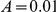. (H): Learning speed when 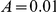. (I): Learning curve when 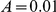.(EPS)

Text S1 **Generality of our results.** This file contains the detailed descriptions of *Generality of our results* section.
(PDF)
