# Supplementary material for: Maximization of Learning Speed in the Motor Cortex Due to Neuronal Redundancy
Source: PLoS Comput Biol. 2012 Jan 12;8(1):e1002348. doi: 10.1371/journal.pcbi.1002348 (PMC3257280; doi:10.1371/journal.pcbi.1002348)
Supplement: Text S1 — Generality of our results. This file contains the detailed descriptions of Generality of our results section. (PDF) [file pcbi.1002348.s006.pdf]

Supporting information for "Maximization of learning speed in the motor cortex due to neuronal redundancy"

Recurrent connections

The motor cortex contains recurrent connections [1], so we needed to confirm that neuronal redundancy maximizes learning speed even when a neural network model includes adaptable recurrent connections. In this case, neural activities are determined by

$$\boldsymbol{A}^t_{k(t)} = (\boldsymbol{I} - \boldsymbol{U}^t)^{-1}\boldsymbol{W}^t\boldsymbol{t}_{k(t)}, \tag{1}$$

where $\boldsymbol{U}^t \in \mathbb{R}^{N\times N}$ represents the recurrent connections in the tth trial. Equation (1) is derived from the stationary solution of the equation $\boldsymbol{A}^t_{k(t)} = \boldsymbol{U}^t\boldsymbol{A}^t_{k(t)} + \boldsymbol{W}^t\boldsymbol{t}_{k(t)}$. $\boldsymbol{U}$ is also learned to minimize the squared error as follows:

$$\boldsymbol{U}^{t+1} = \boldsymbol{U}^t + B_M(\boldsymbol{I} - \boldsymbol{U}^t)^{-1}(\boldsymbol{I} - \boldsymbol{U}^t)^{-1}\boldsymbol{Z}^T\boldsymbol{e}^t\boldsymbol{t}^T_{k(t)}(\boldsymbol{W}^t)^T, \tag{2}$$

where B_M is learning rate.

We numerically calculated learning speed, and figure S1A shows the learning speeds obtained when N=4, 10, 50, or 100, and B_M=0, 0.025, 0.05, 0.075, or 0.1. The whiter the colors, the faster the learning speed. Figures S1B and S1C show the learning curves produced when N=10, 50, or 100 with B_M=0.025 and N=10 with B_M=0, 0.05, or 0.1, respectively. All of the results shown in these figures support our hypothesis that neuronal redundancy maximizes learning speed even when adaptable recurrent connections are included.

Recurrent connections have their own functional roles, however. By comparing the results obtained when $B_M = 0$ and otherwise, we confirmed that recurrent connections facilitate learning speed. Additionally, figure S1D shows the variance of the learning curves when $N = 100$, showing that the more B_M increases, the smaller the variance becomes. This figure suggests another functional role for recurrent connections: recurrent connections not only facilitate learning speed but also increase the stability of learning by decreasing the variance of the learning curve.

Nonlinear activation function

There is nonlinearity in the input-output relationship for neural responses. Therefore, we needed to confirm that neuronal redundancy maximizes learning speed even when the neural network included nonlinearity. In this case, neural activities were determined through a nonlinear function $\sigma(\cdot)$:

$$\boldsymbol{A}^t_{k(t)} = \sigma\big(\boldsymbol{W}^t\boldsymbol{t}_{k(t)}\big), \tag{3}$$

with σ causing the update rule of $\boldsymbol{W}$ to change to

$$\boldsymbol{W}^{t+1} = \boldsymbol{W}^t + B\mathrm{diag}\big(\sigma'(\boldsymbol{W}^t\boldsymbol{t}_{k(t)})\big)\boldsymbol{Z}^T\boldsymbol{R}^T\boldsymbol{e}^t\boldsymbol{t}^T_{k(t)}, \tag{4}$$

where diag($\boldsymbol{a}$) denotes the diagonal matrix in which the (i, i)th component is the ith element of vector $\boldsymbol{a}$, and $\sigma'(\cdot)$ denotes the derivative of the nonlinear function. We use a sigmoid function:

$$\sigma(z) = \frac{K}{2}(1 + \tanh(\beta z)), \tag{5}$$

where K and β indicate the upper limit of the activation function and the slope of the sigmoid function, respectively ($K = 2$ and $\beta = 1$ throughout this section).

Figures S2A and S2B show learning speed when N=10, 50, 100, or 1000 and the learning curve for N=4, 10, or 100, respectively. These figures suggest that neuronal redundancy maximizes learning speed, even in this nonlinear network.

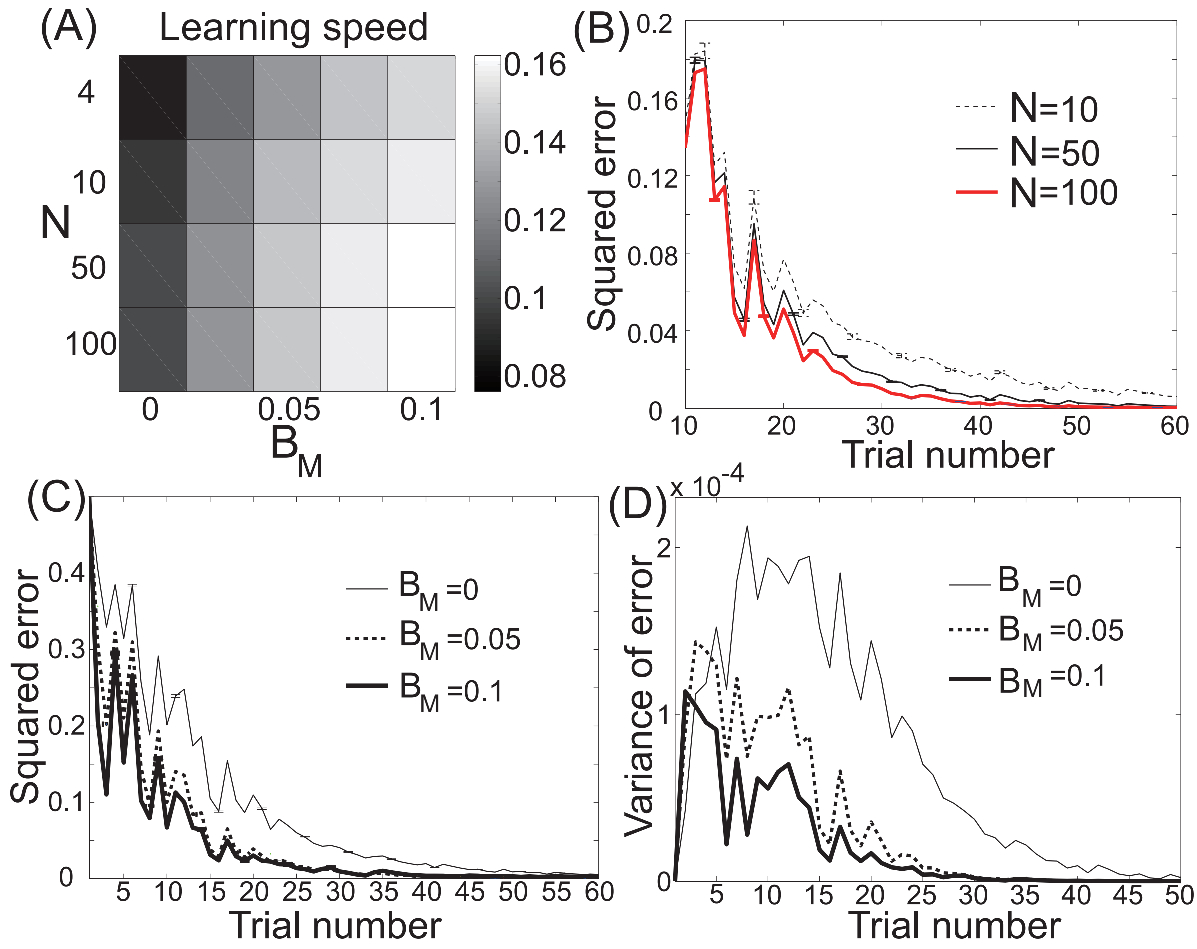

Figure S1: **Relationship between learning speed, neuronal redundancy, and adaptable recurrent connections** ($K = 8$). (A): Learning speed when $N = 4, 10, 50, 100$ and $B_M = 0, 0.025, 0.05, 0.075, 0.1$. The whiter the color, the faster the learning speed. (B): Learning curves obtained when N=10, 50, or 100 and $B_M = 0.025$. These curves show the average values of 1,000 randomly sampled sets of $\boldsymbol{\varphi}$. Error bars represent the standard deviations of the errors. (C): Learning curves obtained when $B_M = 0, 0.05, 0.1$ and $N = 10$. These curves and error bars show average values and standard deviations. (D): Variance of the learning curve when $B_M = 0, 0.05, 0.1$ and $N = 100$ ($K = 8$). These variances are average values from 1,000 randomly sampled sets of $\boldsymbol{\varphi}$.

Nonlinear task

This study has discussed the relationship between neuronal redundancy and learning speed using a linear rotational perturbation $\boldsymbol{R}$, but it is still uncertain whether our results hold when the constrained tasks are nonlinear. In fact, our motor system can solve nonlinear constrained tasks because the system needs to control nonlinear muscle units. We investigated whether our results hold even when a neural network needs to control nonlinear units.

In this case, motor commands are determined by

$$\boldsymbol{x}^t_{k(t)} = \boldsymbol{R}\boldsymbol{Z}_M[\boldsymbol{M}^t_{k(t)}]_+, \tag{6}$$

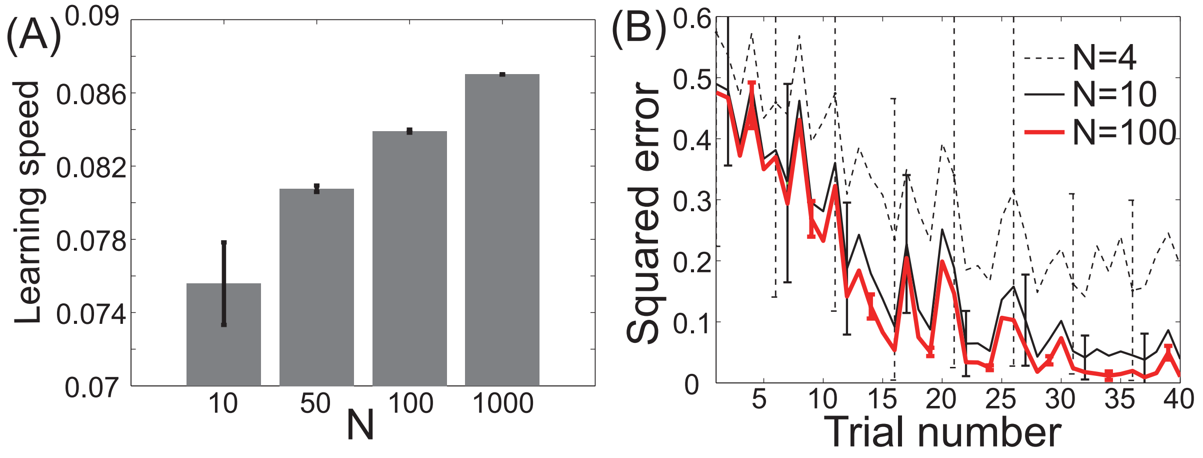

Figure S2: **Relationship between learning speed and neuronal redundancy in the case of a nonlinear neural network ($K = 8$).** (A): Learning speed when N=10, 50, 100, and 1000. The bar graphs and error bars depict sample means and standard deviations, both of which are calculated using the results of 1,000 randomly sampled sets of $\boldsymbol{\varphi}$ values. (B): Learning curves obtained when $N = 4, 10$, or 100. These curves and error bars show average values and the standard deviations of the errors.

where $\boldsymbol{M} \in \mathbb{R}^{Mu \times 1}$ is the activation of muscle units, $\boldsymbol{Z}_{Mu} \in \mathbb{R}^{2\times Mu}$ is the decoder, and Mu represents the number of muscle units. The decoder is defined as $\boldsymbol{Z}_{Mu} = \frac{1}{Mu}\begin{pmatrix} \cos\varphi_1^m & \ldots & \cos\varphi_{Mu}^m \\ \sin\varphi_1^m & \ldots & \sin\varphi_{Mu}^m \end{pmatrix}$, where φ_i^m is the pulling direction of the ith muscle, the direction determined by assuming the wrist step tracking task in a midrange posture [2–4] ($i = 1, ..., M$). $[\cdot]_+$ indicates the threshold linear function that satisfies $[a]_+ = a$ if $a > 0$ and $[a]_+ = 0$ in other cases (this is the function providing nonlinearity). Muscle activations are determined by

$$\boldsymbol{M}_{k(t)}^t = \boldsymbol{C}\boldsymbol{A}_{k(t)}^t, \tag{7}$$

where $\boldsymbol{C} \in \mathbb{R}^{M\times N}$ represents the cortico-muscle connections, and each component is randomly sampled from a normal Gaussian distribution. The synaptic weights $\boldsymbol{W}$ are learned to minimize the squared error between $\boldsymbol{t}$ and $\boldsymbol{x}$:

$$\boldsymbol{W}^{t+1} = \boldsymbol{W}^t + B[\boldsymbol{C}^T]_+\boldsymbol{Z}^T\boldsymbol{R}^T\boldsymbol{e}_{k(t)}^t\boldsymbol{t}_{k(t)}^T, \tag{8}$$

where all elements of the mth row in $[\boldsymbol{C}]_+$ are set to 0 if the mth muscle activity equals 0. Because the nonlinear task is difficult, this task includes 10,000 baseline and 10,000 learning trials.

Figures S3A and S3B show the learning speed when N=10, 20, 50, 100, or 1000 and the learning curve for N=10 or 100. Although there was no significant difference in learning speed between the cases in which N=100 and N=1,000 for the 1% criterion, neuronal redundancy maximized learning speed, even in this nonlinear task.

Stochastic gradient methods

This study used the deterministic gradient descent method, which can explain the results of motor learning experiments. Although recent studies have suggested that this method is likely to be biologically plausible, other studies have suggested that stochastic gradient descent methods are more biologically plausible. We therefore confirmed that our results hold when a stochastic gradient method is used for the learning rule. We considered two representative stochastic methods: the weight perturbation and node perturbation algorithms [5].

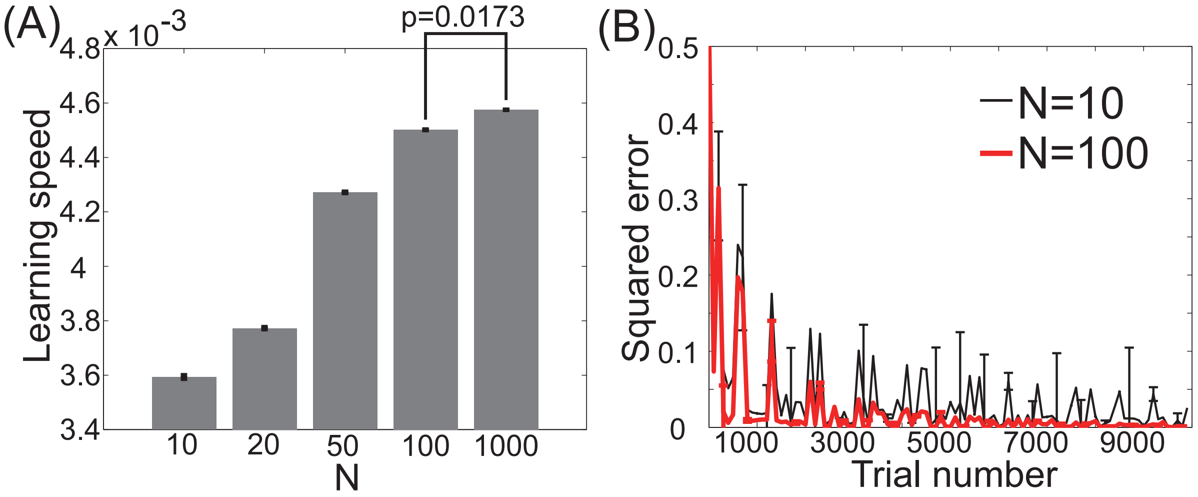

Figure S3: **Relationship between learning speed and neuronal redundancy when the neural network includes nonlinear muscle units** ($K = 8$)**.** (A): The bar graphs and error bars depict sample means and standard deviations, both of which were calculated using the results of 1,000 randomly sampled sets of $\boldsymbol{C}$ values. (B): Learning curves obtained when $N = 10$ or 100. These curves and error bars show average values and the standard deviations of the errors.

Weight perturbation

The weight perturbation algorithm estimates the gradient $\frac{\partial E}{\partial \boldsymbol{W}}$ by applying noise to synaptic weights. Synaptic weight $\boldsymbol{W}$ is updated as

$$\boldsymbol{W}^{t+1} = \boldsymbol{W}^t - \eta\sigma_w(E^t_{WP} - E^t)\boldsymbol{\psi}^t, \tag{9}$$

where $\boldsymbol{\psi}^t \in \mathbb{R}^{N\times 2}$ is a normal Gaussian random variable, σ_w is the standard deviation of the noise ($\sigma_w = 1$ throughout this section), E^t_{WP} is the squared error when the noise $\boldsymbol{\psi}^t$ is applied to synaptic weight $\boldsymbol{W}$, and E^t is the reference error when no noise is applied:

$$E^t_{WP} - E^t = \frac{1}{2}(\boldsymbol{e}^t_{k(t)} - \boldsymbol{R}\boldsymbol{Z}\boldsymbol{\psi}^t\boldsymbol{t}_{k(t)})^T(\boldsymbol{e}^t_{k(t)} - \boldsymbol{R}\boldsymbol{Z}\boldsymbol{\psi}^t\boldsymbol{t}_{k(t)}) - \frac{1}{2}(\boldsymbol{e}^t)^T_{k(t)}\boldsymbol{e}^t_{k(t)}. \tag{10}$$

Noise averaging yields the update rule of synaptic weights:

$$\langle \boldsymbol{W}^{t+1}\rangle = \langle \boldsymbol{W}^t\rangle + B\sigma_w^2\boldsymbol{Z}^T\boldsymbol{R}^T\boldsymbol{e}^t_{k(t)}\boldsymbol{t}^T_{k(t)}. \tag{11}$$

This is equivalent to deterministic gradient descent, except for the noise variance σ_w^2. Because of this equivalence, neuronal redundancy also maximizes learning speed when the learning rule is weight perturbation.

We confirmed these analytical results using numerical simulations. Figures S4A and S4B show the learning speed when N=10, 50, 100, or 1,000 and the learning curve when N=4, 10, or 100 when the weight perturbation is used. Although there was no significant difference in learning speed between the cases in which N=100 and N=1,000, neuronal redundancy maximized learning speed, even in the case of weight perturbation.

Node perturbation

Node perturbation estimates the gradient $\frac{\partial E}{\partial \boldsymbol{W}}$ by applying noise to the output units. In the algorithm, the update rule of the synaptic weight $\boldsymbol{W}$ is

$$\boldsymbol{W}^{t+1} = \boldsymbol{W}^{t} - \boldsymbol{Z}^{T}\boldsymbol{R}^{T}\eta\sigma_n(E^t_{NP} - E^t)\boldsymbol{\epsilon}^t\boldsymbol{t}^T_{k(t)}, \tag{12}$$

where $\boldsymbol{\epsilon}^t \in \mathbb{R}^{2\times 1}$ is a normal Gaussian random variable, σ_n is the standard deviation of the noise ($\sigma_n = 0.3$ throughout this study), E^t_{NP} is the squared error when the noise $\boldsymbol{\epsilon}^t$ is applied to the output units, and E^t is the reference error when no noise is applied:

$$E^t_{NP} - E^t = \frac{1}{2}(\boldsymbol{t}_{k(t)} - (\boldsymbol{x}_{k(t)} + \boldsymbol{\epsilon}^t))^T(\boldsymbol{t}_{k(t)} - (\boldsymbol{x}_{k(t)} + \boldsymbol{\epsilon}^t)) - \frac{1}{2}(\boldsymbol{t}_{k(t)} - \boldsymbol{x}_{k(t)})^T(\boldsymbol{t}_{k(t)} - \boldsymbol{x}_{k(t)}). \tag{13}$$

Noise averaging yields the following update rule for synaptic weights:

$$\langle \boldsymbol{W}^{t+1} \rangle = \langle \boldsymbol{W}^{t} \rangle + B\sigma_n^2\boldsymbol{Z}\boldsymbol{R}\boldsymbol{e}^t_{k(t)}\boldsymbol{t}^T_{k(t)}. \tag{14}$$

This is equivalent to deterministic gradient descent, except for noise variance σ_n^2. Because of this equivalence, neuronal redundancy also maximizes learning speed when node perturbation is used as the learning rule.

Figures S4C and S4D show the learning speed when N=10, 50, 100, or 1000 and the learning curve for N=4, 10, or 100 when node perturbation is used. Although there was no significant difference in learning speed between the cases in which N=100 and N=1,000, neuronal redundancy maximized learning speed, even in the case of node perturbation.

Synaptic decay

As described in the *Methods* section, when synaptic decay exists ($0 \ll A < 1$), our analytical calculations revealed that neuronal redundancy not only maximizes learning speed but also minimizes residual error. We confirmed this analytical result using numerical simulations. Figures S5A, S5D, and S5G show the residual error when $A = 0, 0.005$, and 0.01, respectively. Figures S5B, S5E, and S5H show learning speed when $A = 0, 0.005$, and 0.01, respectively. The results in these figures confirm our analytical results; neuronal redundancy not only maximizes learning speed but also minimizes residual error.

Optimal learning rate

We defined the learning rate as NB, but it remains unclear why the rate is multiplied by N. Werfel et al. reported that the optimal learning rate and speed decrease as the number of neurons increases [5], which is contradictory to our results. It should be noted that, in their model, the number of neurons N corresponds to the number of input units M. This section analytically reveals that learning rate should be $\mathcal{O}(N)$ and that our results do not contradict Werfel's results. In our study, the optimal learning rate is $\mathcal{O}(N)$, and learning speed is inversely proportional to the number of input units.

To analytically calculate the optimal learning rate and speed, each component of $\boldsymbol{t}$ is assumed to be randomly sampled from a Gaussian distribution whose mean and variance are 0 and γ^2, respectively. The optimal learning speed ideally should be investigated using all of the possible input sequences $\boldsymbol{t}^1$, ..., $\boldsymbol{t}^{Trial}$. Although, in general, this calculation is analytically and numerically intractable, we can calculate the average learning speed across all the possible input sequences by calculating the average learning curve

$$\langle E^{t+1} \rangle = \int d\boldsymbol{t}P(\boldsymbol{t})E^{t+1} \tag{15}$$

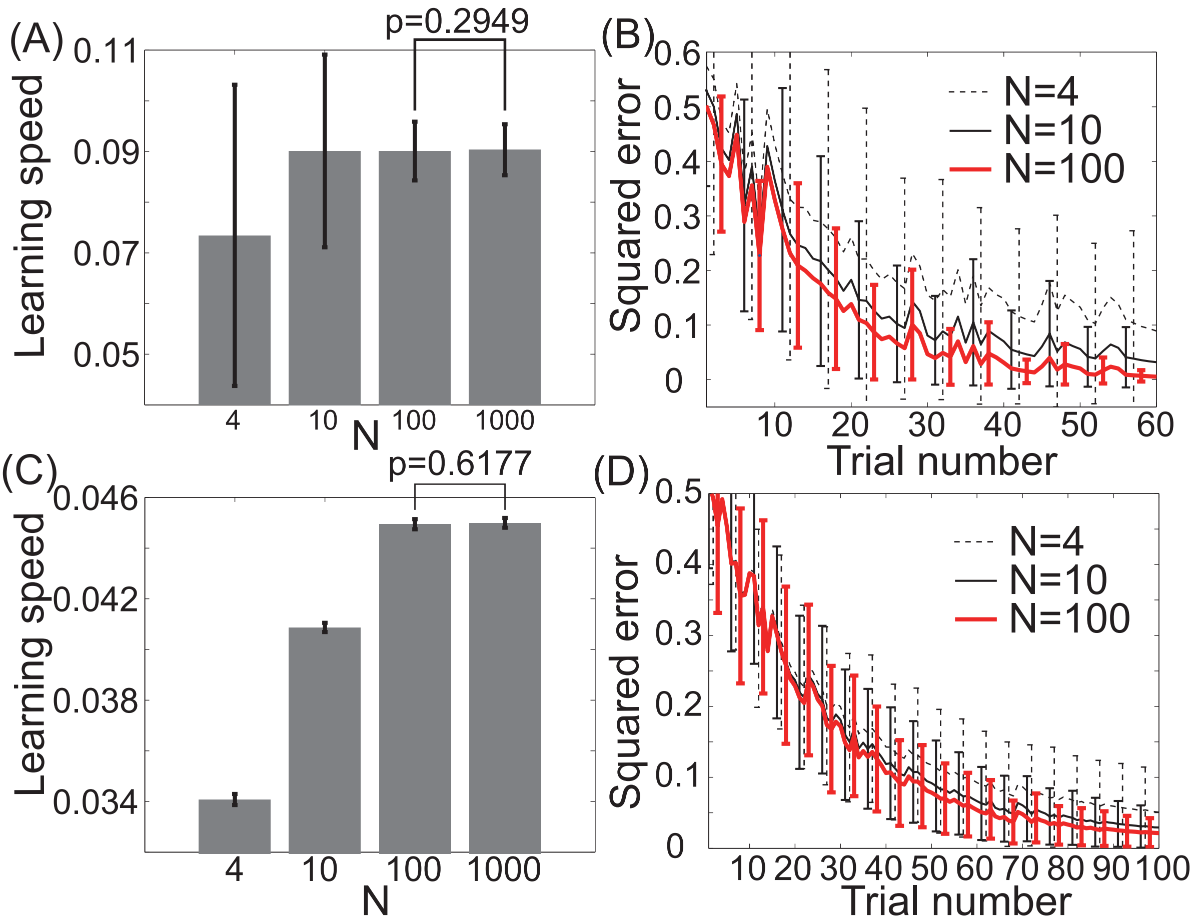

Figure S4: **Relationship between learning speed and neuronal redundancy in the case of weight perturbation and node perturbation ($K = 8$).** (A): Learning speed when $N = 4, 10, 100$, or 1000, with weight perturbation as the learning rule. The bar graphs and error bars depict sample means and standard deviations, both of which are calculated using the results of 1,000 randomly sampled sets of φ. (B): Learning curves obtained when $N = 4, 10$, or 100, with weight perturbation as the learning rule. These curves and error bars show the average values and the standard deviations of the errors. (C): Learning speed when $N = 4, 10, 100$, or 1000, with node perturbation as the learning rule. The bar graphs and error bars depict sample means and standard deviations, both of which are calculated using the results of 1,000 randomly sampled sets of φ. (D): Learning curves obtained when $N = 4, 10$, or 100, with node perturbation as the learning rule. These curves and error bars show average values and the standard deviations of the errors.

if $P(\boldsymbol{t})$ is Gaussian. Equation (15) can be written as

$$\begin{aligned}\langle E^{t+1}\rangle &= \frac{1}{2}\int d\boldsymbol{t}^{\boldsymbol{t+1}}P(\boldsymbol{t}^{\boldsymbol{t+1}})d\boldsymbol{t}^{\boldsymbol{t}}P(\boldsymbol{t}^{\boldsymbol{t}})(\boldsymbol{t}^{t+1})(\boldsymbol{I}-\boldsymbol{Z}\boldsymbol{W}^{t+1})^T(\boldsymbol{I}-\boldsymbol{Z}\boldsymbol{W}^{t+1})\boldsymbol{t}^{t+1} \\ &= \frac{\gamma^2}{2}\mathrm{Tr}\big[(\boldsymbol{I}-\boldsymbol{Z}\boldsymbol{W}^{t+1})^T(\boldsymbol{I}-\boldsymbol{Z}\boldsymbol{W}^{t+1})\big], \qquad (16)\end{aligned}$$

where we use $\boldsymbol{x}^T\boldsymbol{A}\boldsymbol{x} = \mathrm{Tr}[\boldsymbol{A}\boldsymbol{x}\boldsymbol{x}^T]$. Substituting the update rule of $\boldsymbol{W}^{t+1}$, equation (16) can be written

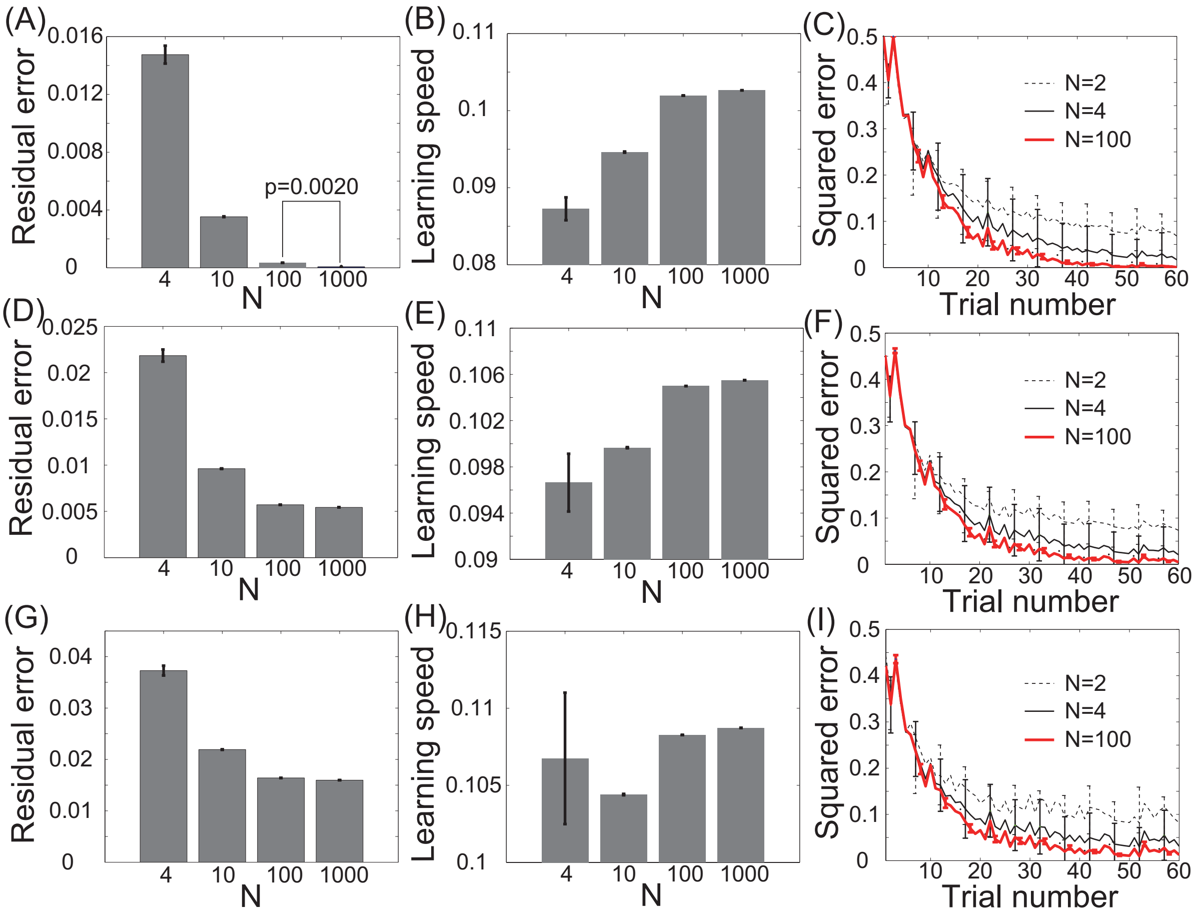

Figure S5: **Relationship between residual error, learning speed, and neuronal redundancy with synaptic decay included ($K = 8$).** (A): Residual error when $A = 0$. The bar graphs and error bars denote sample means and standard deviations, both of which are calculated using the results of 1,000 randomly sampled sets of φ values. (B): Learning speed when $A = 0$. The bar graphs and error bars depict sample means and standard deviations. (C): Learning curves obtained when $N = 4, 10$, and 100 and $A = 0$. These curves and error bars show average values and standard deviations. (D): Residual error when $A = 0.005$. (E): Learning speed when $A = 0.005$. (F): Learning curve when $A = 0.005$. (G): Residual error when $A = 0.01$. (H): Learning speed when $A = 0.01$. (I): Learning curve when $A = 0.01$.

as

$$\begin{aligned}\langle E^{t+1}\rangle &= \frac{\gamma^2}{2}\mathrm{Tr}\big[(\boldsymbol{I}-\boldsymbol{Z}\boldsymbol{W}^t)^T(\boldsymbol{I}-\boldsymbol{Z}\boldsymbol{W}^t) - 2\eta\gamma^2(\boldsymbol{I}-\boldsymbol{Z}\boldsymbol{W}^t)^T\boldsymbol{Z}\boldsymbol{Z}^T(\boldsymbol{I}-\boldsymbol{Z}\boldsymbol{W}^t) \\ &+ 2\eta^2\gamma^4(\boldsymbol{I}-\boldsymbol{Z}\boldsymbol{W}^t)^T\boldsymbol{Z}\boldsymbol{Z}^T\boldsymbol{Z}\boldsymbol{Z}^T(\boldsymbol{I}-\boldsymbol{Z}\boldsymbol{W}^t) + \eta^2\gamma^4\mathrm{Tr}[(\boldsymbol{I}-\boldsymbol{Z}\boldsymbol{W}^t)^T\boldsymbol{Z}\boldsymbol{Z}^T\boldsymbol{Z}\boldsymbol{Z}^T(\boldsymbol{I}-\boldsymbol{Z}\boldsymbol{W}^t)]\big],\end{aligned} \tag{17}$$

which can be simplified as

$$(\bar{e}_{km}^{t+1})^2 = \gamma^2(1-2\eta\gamma^2\lambda_k + (M+2)\eta^2\gamma^4\lambda_k^2)(\tilde{\bar{e}}_{km}^t)^2 = L_k(\eta)(\bar{e}_{km}^t)^2, \tag{18}$$

where we use $\langle \boldsymbol{t}\boldsymbol{t}^T\boldsymbol{A}\boldsymbol{t}\boldsymbol{t}^T\rangle = \gamma^4(\boldsymbol{A}+\boldsymbol{A}^T) + \gamma^4\mathrm{Tr}[\boldsymbol{A}]\boldsymbol{I}$, $\boldsymbol{Z}^T\boldsymbol{Z} = \boldsymbol{V}^T\boldsymbol{\lambda}\boldsymbol{V}$, $\bar{\boldsymbol{e}} = \boldsymbol{V}(\boldsymbol{I}-\boldsymbol{Z}\boldsymbol{W})$, λ_k is the kth

eigenvalue of $\boldsymbol{Z}^T\boldsymbol{Z}$ and $\mathcal{O}(\frac{1}{N})$, and λ_k^2 is $\mathcal{O}(\frac{1}{N^2})$ $(k = 1, ..., M,\ m = 1, ..., M)$. Learning speed is thus determined by $L_k(\eta)$ because

$$\langle E^{t+1}\rangle = \frac{\gamma^2}{2}\sum_k^M\sum_m^M(\bar{e}_{km}^{t+1})^2 = \frac{\gamma^2}{2}\sum_k^M\sum_m^M L_k(\eta)(\bar{e}_{km}^{t})^2, \tag{19}$$

which means that, the smaller L_k becomes, the faster the learning speed becomes.

We can calculate the maximal learning speed with optimization of $L_k(\eta)$ with respect to η. We define the cost function as the average learning speed

$$L(\eta) = \frac{1}{M}\sum_k^M L_k(\eta) = \frac{1}{M}\sum_k^M \gamma^2(1 - 2\eta\gamma^2\lambda_k + (M+2)\eta^2\gamma^4\lambda_k^2) \tag{20}$$

because k-independent η needs to be determined to minimize all the $L_k(\eta)$. Optimized η can be calculated as

$$\eta^* = \frac{1}{\gamma^4(M+2)}\frac{\sum_k^M \lambda_k}{\sum_k^M \lambda_k^2} \tag{21}$$

which means the optimized $L(\eta^*)$ is given by

$$L(\eta^*) = 1 - \frac{1}{M}\frac{1}{M+2}\frac{(\sum_k^M \lambda_k)^2}{\sum_k^M \lambda_k^2}. \tag{22}$$

Thus, learning speed decreases if $M(=T)$ increases, which is in agreement with Werfel's result. Numerical calculations support this agreement and our hypothesis: the more M grows, the slower learning speed becomes, and the more N grows, the faster learning speed becomes (figure S6A). In addition, when each component of $\boldsymbol{Z}$ is $\mathcal{O}(\frac{1}{N})$, η^* is $\mathcal{O}(\frac{N}{M})$, which means that the optimal learning rate can be written as $\eta^* = NB^*$. It should be noted that neuronal redundancy maximizes learning speed even when each component of $\boldsymbol{Z}$ is $\mathcal{O}(1)$ (figure S6B). In this case, η^* is $\mathcal{O}(\frac{1}{N})$. This study therefore considered a near-optimal learning rate, and our results do not contradict to Werfel's study.

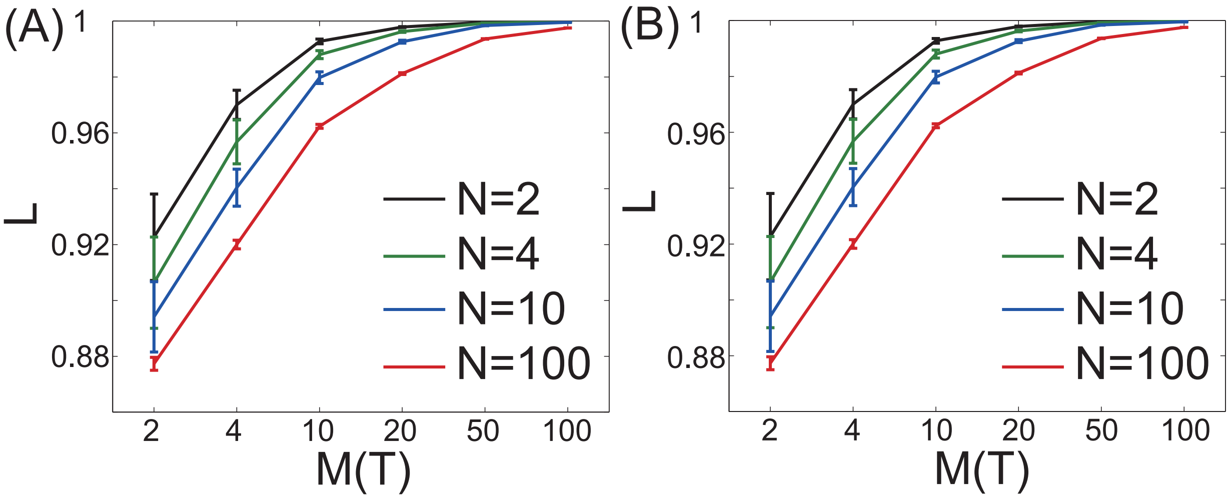

Figure S6: **Calculated** $L(\eta^*)$ **values for** $N = 2, 4, 10,$ **or** 100 **and** $T = 1, 2, 4, 10, 20, 50,$ **or** 100**.** (A): Solid lines and error bars depict means and standard deviations of the values calculated using 100 sets of randomly sampled $\boldsymbol{Z}$ values. Z_{ji} has a mean and variance of 0 and $\frac{1}{N^2}$, respectively. The smaller $L(\eta^*)$ becomes, the faster learning speed becomes. (B): Calculated $L(\eta^*)$ values obtained when each component of $\boldsymbol{Z}$ is $\mathcal{O}(1)$, i.e., Z_{ji} has mean and variance that are 0 and 1, respectively.

References

1. Capaday C, Ethier C, Brizzi L, Sik A, van Vreewijk C, Gingras D. (2009) On the nature of the intrinsic connectivity of the cat motor cortex: evidence for a recurrent neural network topology. *J. Neurophysiol.* **102**: 2131-2141.

2. Hoffman D, Strick P, (1999) Step-tracking movements of the wrist. IV. Muscle activity associated with movements in different directions. *J. Neurophysiol.* **81**(1): 319-333.

3. Fagg A, Shah A, Barto A, (2002) A computational model of muscle recruitment for wrist movements. *J. Neurophysiol.* **88**(6): 3348-3358.

4. Shah A, Fagg A, Barto A, (2004) Cortical involvement in the recruitment of wrist muscles. *J. Neurophysiol.* **91**(6): 2445-2456.

5. Werfel J, Xie X, Seung S, (2005) Learning curves for stochastic gradient descent in linear feedforward networks. *Neural Compt.* **17**(12): 2699-2718.
